# Structure-Acid Lability Relationship of *N*-alkylated α,α-dialkylglycine Obtained via a Ugi Multicomponent Reaction

**DOI:** 10.3390/molecules26010197

**Published:** 2021-01-02

**Authors:** Iván Ramos-Tomillero, Marisa K. Sánchez, Hortensia Rodríguez, Fernando Albericio

**Affiliations:** 1 Institute for Research in Biomedicine, Barcelona Science Park, 08028 Barcelona, Spain; ivan.ramostomillero@radboudumc.nl; 2 CIBER-BBN, Networking Centre on Bioengineering, Biomaterials and Nanomedicine, Department of Organic Chemistry, University of Barcelona, 08028 Barcelona, Spain; 3 School of Chemical Sciences and Engineering, Yachay Tech University, Yachay City of Knowledge, Urcuqui 100650, Ecuador; marisa.sanchez@yachaytech.edu.ec; 4 Institute for Advanced Chemistry of Catalonia (IQAC-CSIC), 08034 Barcelona, Spain; 5 School of Chemistry and Physics, University of KwaZulu-Natal, Durban 4001, South Africa

**Keywords:** *N*-alkylated α,α-dialkylglycine, Ugi multicomponent reaction, structure-acid lability, acidolysis study

## Abstract

Using the classical Ugi four-component reaction to fuse an amine, ketone, carboxylic acid, and isocyanide, here we prepared a short library of *N*-alkylated α,α-dialkylglycine derivatives. Due to the polyfunctionality of the dipeptidic scaffold, this highly steric hindered system shows an interesting acidolytic cleavage of the C-terminal amide. In this regard, we studied the structure-acid lability relationship of the C-terminal amide bond (cyclohexylamide) of *N*-alkylated α,α-dialkylglycine amides **1a–n** in acidic media and, afterward, it was established that the most important structural features related to its cleavage. Then, it was demonstrated that electron-donating effects in the aromatic amines, flexible acyl chains (Gly) at the *N*-terminal and the introduction of cyclic compounds into dipeptide scaffolds, increased the rate of acidolysis. All these effects are related to the ease with which the oxazolonium ion intermediate forms and they promote the proximity of the central carbonyl group to the C-terminal amide, resulting in C-terminal amide cleavage. Consequently, these findings could be applied for the design of new protecting groups, handles for solid-phase synthesis, and linkers for conjugation, due to its easily modulable and the fact that it allows to fine tune its acid-lability.

## 1. Introduction

The classical Ugi four-component reaction (U-4CR) [1,2] is an isocyanide-based multicomponent reaction (IMCR) in which an amine, a carbonyl compound (ketone), a carboxylic acid, and an isocyanide render *N*-alkylated α,α-dialkylglycine derivatives. The U-4CR has been widely used in heterocyclic chemistry, [3,4] in modern synthetic chemistry [5,6] and for ligation and bioconjugation [7]. It has also found ample applications for macrocyclization and stapling peptides, [8] the production of pneumococcal multivalent glycoconjugates as vaccine candidates [9], the stabilization of cyclic β‑hairpins [10], and even in the total synthesis of exigurin, which is a natural marine product [11]. 

*N*-alkylated α,α-dialkylglycine derivatives are characterized by considerable steric hindrance around the two amides, thereby jeopardizing their synthesis in solution and in solid-phase. In this context, the U-4CR can be used to prepare these compounds, which, in an acidic medium, are converted into labile systems. Although Ugi products have a broad range of synthetic applications, only a few studies have addressed the acidolytic behavior of these structures. In 1999, Gootman et al. [12] reported an unusual amide bond scission in acidic media in derivatives containing acylated *N*-methyl-α-aminoisobutyryl (*N*MeAib) residues. Through X-ray diffraction studies, they found that the carbonyl oxygen atom of the vicinal residue in the sequence is closer to the carbonyl carbon of the *N*MeAib residue and that it acts as an internal nucleophile, promoting a cleavage reaction via a postulated oxazolinium ion intermediate.

In 2003, Maia et al. [13] reported the synthesis and acidolytic behavior of several α,α-dialkyl glycine derivatives obtained by the Ugi–Passerini reaction. They realized that neither the alkyl groups of the α,α-dialkylated glycine nor the nature of the starting isonitrile influences the ease of cleavage of the C-terminal amide bond. Additionally, using five *N*-alkylated α,α-dialkylglycine derivatives, they concluded that acidolysis was less affected by steric effects than expected [14].

**Figure 1:**
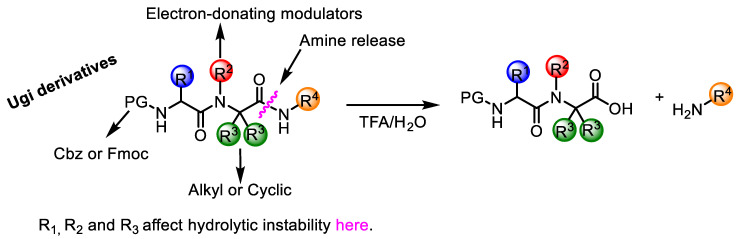
Patterns of *N*-alkylated α,α-dialkylglycine and acidolysis of the C-terminal amide bond of α,α-dialkylglycine derivatives and their dependence on R1 and R2.

Later, the same authors studied the synthesis in greater depth by means of a Ugi–Passerini reaction and examined the acidolysis of *N*-phenylacetyl-*N*-benzyl-α,α-dimethylglycine cyclohexylamide derivatives and their α,α-dibenzylglycine analogs. They concluded that the acidolysis of the C-terminal amide bond depends mostly on polar substituent parameters rather than steric. In addition, they achieved the isolation of an oxazolone derivative, thereby confirming Goodman’s proposal and demonstrating that amide cleavage proceeds through a cyclic intermediate [15]. A similar behavior was also observed for *N*-acylated peptoids, which trigger the loss of peptoid monomer from the sequence, a phenomenon also explained through an oxazolinium-based mechanism [16]. Furthermore, the aforementioned acid-cleavable behavior of α,α-dialkylglycines has also been used to carry out the synthesis of tri- and pentapeptides with a central residue of one α,α-dialkylglycine [17]. Those authors demonstrated that the overall yields of the peptides was significantly related to molecular bulkiness around the reaction center (R2) rather than to the size of the peptide chain involved. In addition, Scott et al. demonstrated that the hydrolytic instability of *N*-acylated amino acid amides depends on the mono-substituted aromatic acyl groups (R1CO), four bonds away from the site of hydrolysis—a process accelerated by electron-rich substituents. Therefore, the acidolysis of the C-terminal amide bond of α,α-dialkyl glycine derivatives is dependent on both R1 and R2 (Figure 1). Furthermore, the effect of temperature on acidolysis as well as the influence of the nature of the substituents [18] has also been studied, showing that changes in the reaction rate are governed by variations in both the enthalpy and entropy of activation, which are related to bond energy and steric hindrance, respectively [19]. 

Taking into consideration the acid-labile properties of this scaffold, our group has recently designed a small library (14 members) of *N*-alkylated α,α-dialkyl glycine derivatives prepared via the U-4CR (Figure 1). This system allows the release of the C-terminal amide bond.

## 2. Results and Discussion

**Figure 2:**
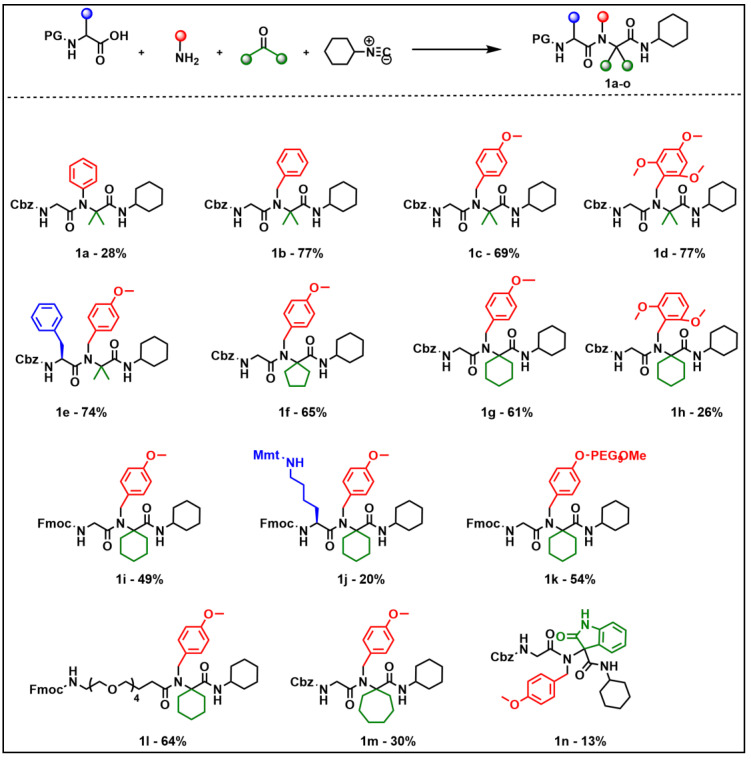
The U-4CR to yield *N*-alkylated α,α-dialkylglycine (**1a**–**n**).

The *N*-alkylated α,α-dialkylglycine derivatives were synthesized by means of the U-4CR using a primary amine, a ketone, a carboxylic acid, and cyclohexyl isocyanide (Figure 2). The primary amine provided the system with the *N*-alkyl substituent, the ketone the two α,α-substituents, and the carboxylic acid, which is an α-amino acid, the acyl group. In addition, the α-amino is protected either with the fluorenylmethoxycarbonyl (Fmoc) or the carbobenzoxy (Cbz) groups, and upon removal of the protecting group the *N*-terminal amine can be used for a further modification. This outstanding methodology allows the preparation of complex systems, in most cases with satisfactory yields and reaction times ranging from overnight to three weeks (Figure 2).

The reactions were carried out using an excess of the starting alkyl or cyclic ketone, which also acts as a solvent. For high boiling point or solid ketones, it was necessary to add small amounts of methanol (MeOH) to the reaction mixture. In addition to acetone, we used cyclic ketones of five, six, and seven members and the isatin as a particular case. Moreover, Cbz- or Fmoc-Gly-OH, Cbz-Phe-OH, Cbz-Ala-OH, Fmoc-Lys(Mtt)-OH (Mtt, 4-methyltrityl), or Fmoc-NH-(PEG)_4_-OH (PEG, polyethylenglycol) were used as carboxylic acid. The presence of the PEG on the last amino acid derivative served to increase the solubility of the Ugi construct. The presence of these amino acids allows further derivatization of the final product through the α-amino function. In the case of the Lys derivative, the α-amino group can serve as an additional derivatization point.

The starting amines were commercially available, with the exception of 4-MeO-PEG_9_-benzylamine, which was prepared by the reaction of the 4-cyanophenol and bromo-PEGylated derivative, followed by nitrile reduction. Furthermore, a PEG-based amine was once again used to increase the solubility of the final Ugi construct. In addition, all the amines used were benzylamines, except aniline. Some of the benzylamines have electron-donating groups (metoxy), which is an important factor for the study of the lability of the Ugi product since the bulkiness around the nitrogen of the amine is related to C-terminal amide bond cleavage.

According to Costa et al. [13] the substituent of the C-terminal amide does not play an important role in its release. Therefore, cyclohexyl isocyanide was chosen based solely on its availability and price. Experimental details of the syntheses and full characterization have been described earlier [20]. All Ugi reactions furnished the target products (**1a**–**n**) (Figure 2) from low (13%) to high yields (77%) and without evidence of amino acid racemization for Phe and Lys (**1e**, **1j**) (yields were not optimized). 

To study the lability of the C-terminal amide bond (cyclohexylamide) in acidic media and, therefore, to define the most important structural features related to its cleavage, the *N*-alkylated α,α-dialkylglycine amides **1a**–**n** were treated with different concentration of acids until complete amine release (Figure 2, Table 1).

**Figure 3:**
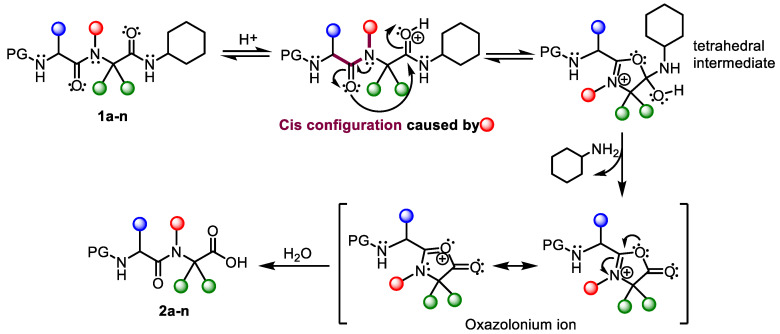
Acidolysis of *N*-alkylated α,α-dialkylglycine (**1a**–**n**) to give (**2a**–**n**) and the corresponding C-terminal amide cleavage reaction mechanism via oxazolonium ion intermediate.

To study the rate of acidolysis, we introduced modifications into the components of α,α-dialkylglycinamide in order to modulate the acidolytic process. First, various amines were tested to determine the effect of their electron-donating groups on C-terminal amine release. The α,α-dialkylglycines derived from aniline, benzyl-, 4-metoxybenzyl-, and 2,4,6-trimethoxybenzylamine using Cbz-Gly-OH and acetone were synthesized (**1a**–**d**). As expected, the low nucleophilicity of aniline (**1a**) resulted in a lower reaction yield (28% in 12 days) than benzyl amines (**1b**–**d**), which gave a yield around 70% (15–21 days) in the three cases. On the other hand, 4-methoxybenzylamine (**1g**), which had the same Cbz-Gly-OH and cyclohexanone, gave a yield of 61% in five days. The reduced yield (28%) for compound **1h** may be attributable to the presence of the methoxy groups in positions 2 and 6. However, the results showed superior yields when the 2,4,6-trimethoxybenzylamine (**1d**) was used. No explanation has been found to explain this observation. Second, when the carboxylic acid was analyzed, the Cbz group (**1g**) gave a greater yield than the Fmoc group (**1i**), and the participation of amino acids with the side-chain [Lys(Mtt) (**1j**)] also had a negative impact on yield, as expected due to the hindrance around the carboxylic group. In contrast, 1e, which contains Phe, an amino acid amenable to coupling, rendered a good reaction yield. Third, regarding the ketones, the α,α-dialkyl amino acid moiety, dimethyl (**1c**), cyclopentyl (**1f**), and cyclohexyl (**1g**) gave similar results. Finally, when the isatin was used (**1n**), a very low yield was obtained, accompanied by a side product. To assess the selectivity of the amide bond cleavage in acidic media, Ugi derivatives **1a**–**n** were treated with acid at a range of concentrations until completion of amine release. However, some amines were not fully hydrolyzed (Figure 3 and Table 1). The lack of solubility of the protected dipeptides hindered the study of acidolysis in aqueous solvents. In this regard, previous work was done using acetonitrile (MeCN) to solubilize the protected dipeptides, thus determining the compounds with a higher rate of acidolysis. Furthermore, we tested H_2_O/MeCN mixtures and observed that the hydrolysis rate was affected. The experiments were performed by treating a solution of the amidated compounds **1a**–**n** in MeCN (1 mg/mL) with 1% of trifluoroacetic acid (TFA), and the acidolytic reactions were monitored directly by HPLC at various time points (Table 1) (Figure 3). Moreover, the main product from the acidolysis (carboxylic acid derivatives) was identified through mass spectrometry (See SI).

It is well established that acidolysis occurs via an intramolecular tetrahedral intermediate, followed by the formation of the oxazolinium ion (Figure 3). Once the tetrahedral intermediate has formed, the lone-pair electrons on the nitrogen of cyclohexyl are no longer in conjugation with the carbonyl π-bond, and it becomes a proton acceptor. Thus, the amine is released and an oxazinium ion intermediate is formed. This intermediate reacts with trace water to form the carboxylic acid product. 

**Figure 4:**
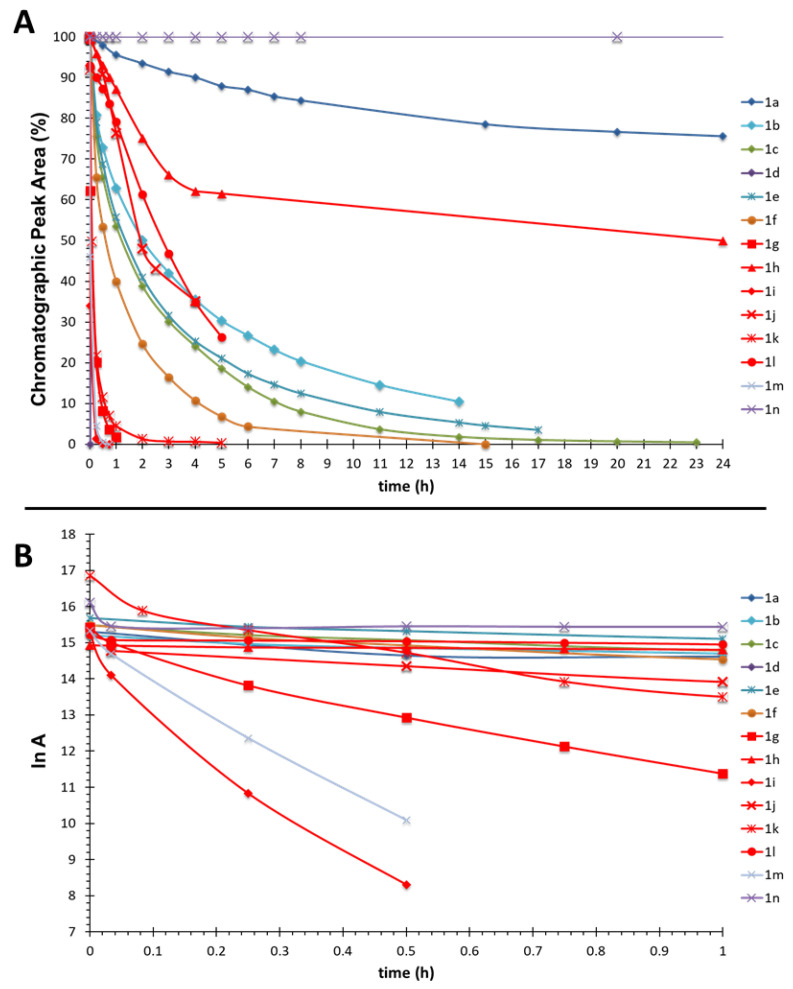
Acidolysis study for compounds **1a**-**n** at 1 mg/mL in TFA/MeCN (1:99). (**A**) Chromatographic peak area (%) corresponds to amide degradation at the indicated time and (**B**) linearization of the acidolysis curve, A corresponds to the chromatographic peak area of the amides **1a**–**n** at the indicated time of treatment.

Accordingly, acyclic and cyclic ketones were used to synthesize the Ugi adducts, to restrict the conformation around the dialkylglycine and thus, to analyze the role of these substituents, as well the nitrogen of the carboxamide. Almost all the compounds studied underwent C-terminal amide acidolysis with the release of the amine (Table 1) and the formation of the corresponding carboxylic acid from high (**1a**) to short (**1d**, **1i**, and **1m**) reaction times (Figure 4A). The faster reactions (compounds **1i**, **1m**, and **1g**) showed pseudo-first-order behavior with respect to the amide derivative, as reflected by the linear representation of ln A, being A HPLC peak area, in front of time (Figure 4B). In addition, most logarithmic graphs of unimolecular reaction rates are linear, with regression values of 0.977 at least (Figure 4B).

The *N*-substituent of the carboxamide drives amide adoption of a *cis* configuration, which should facilitate cyclization (Figure 3). Furthermore, the presence of electron-donating groups in that substituent should favor the attack of the first amide oxygen. By conferring the amine electronic density, these groups weaken the CO-N bond since *N* has electronic density, and there is not enough attraction between the CO-N bond, thereby enhancing amine release. A comparison of compounds **1a**–**d**, in which the only difference is the *N*-substituent, revealed that the rate of acidolysis rate rose as the presence of electron-donating groups increased. In this regard, **1d**, which contains the trimethoxybenzyl group, showed greater amide acidolysis than **1c** and **1b**, which contain p-methoxybenzyl and benzyl groups, respectively. However, the reactivity **1d** to TFA-MeCN was so high that it led to complete compound decomposition, since treatment of the adducts with 95% TFA caused cleavage of the *N*-alkylated benzyl. The addition of water to the reaction mixture led to a decrease in the rate of acidolysis, as previously described for other examples of amide scission [12]. 

Additionally, we observed that the *N*-terminal amino acid side chain affected the rate of acidolysis (see compound **1c** vs. **1e** and **1i** vs. **1j**). Accordingly, the flexibility and the major tendency of the system to adopt a cis conformation conferred by Gly facilitates the formation of the oxazolone ring intermediate, thereby allowing cleavage of the C-terminal amide.

Going a step further, the introduction of bulky and steric restriction groups in the α-carbon of the dialkylglycine significantly enhanced amide acidolysis. Hence, the presence of a cyclic motif directly affected the formation of the oxazolinium ion, thereby increasing the amide cleavage rate. The decreasing differences in terms of the acidolysis rate observed between the acyclic (dimethyl) derivative (**1c**, t_1/2_ = 3 h) and the corresponding penta-, hexa- and heptacyclic derivatives (**1f**, **1g** and **1m** t_1/2_ = 1.5, 0.2 and 0.1 h, respectively) reveal the cyclic Ugi derivatives as best hydrolyzable compounds. Correspondingly, the introduction of cyclic compounds into the dipeptide scaffolds leads to the approximation of the central carbonyl group to the C-terminal amide through an angle reduction, and consequently, acidolysis occurs faster than with the acyclic compounds. Finally, the synthesized isatin derivative, whose behavior was expected to be similar to that of cyclic derivatives, was stable under the acidolytic conditions tested. 

As the introduction of a large PEG chain into the protected dipeptide (**1k**) improved the water solubility of the fully protected dipeptides, the acidolysis of compound **1k** was also tested using milder acid conditions in aqueous media. To this end, **1k** was treated with an acidolytic cocktail that is commonly used to mimic the interior of lysosomes. The mixture contained 2-(*N*-morpholino)ethanesulfonic acid (MES) (0.1 M), NaCl (137 mM) and KCl (2.7 mM) at pH = 4.8. Compound **1k** was incubated in the mentioned buffer at 37 °C for 24 h. Unfortunately, it remained stable under these conditions. This stability could jeopardize the use of such systems as cleavable linkers for bioconjugation since they would remain stable at the lower pH conditions (4.8) found in systemic circulation. Generally, cyclo-containing Ugi compounds (**1f**–**m**) showed a higher acidolysis rate than the acyclic adducts (**1c** and **1e**).

## 3. Materials and Methods

### 3.1. General Methods

The acidolysis study was carried out treating the protected amides **1a–n** (1 mg/mL in MeCN) with 1% of trifluoroacetic acid (TFA). When is indicated, the acidolysis was carried out in H_2_O/MeCN (1:1). The reaction was performed in sealed HPLC-glass vials of 2 mL, analyzing the mixture from 2 min to 24 h and the kinetic overlay HPLC chromatograms corresponding to each study are showed below. Only compounds **2c**, **2e**, **2g**, **2i–l** were isolated by the indicated technique for further studies. The isolation was carried out by first removing the solvent under reduced pressure. Then the solid was dissolved in DCM (40 mL) and washed with HCl 0.1 N (3 × 30 mL) and water (3 × 30 mL). Then, the organic layer was dried over MgSO_4_, filtered and the solvent was removed under reduced pressure to yield compounds **2c**, **2e**, **2g**, **2i–l**.

### 3.2. Acidolysis of ***1a**–**n*** Compounds via HPLC

#### 3.2.1. Acidolysis of **1a**

RP-HPLC linear gradients of H_2_O/MeCN (95:5) to (0:100) over 8 min.

#### 3.2.2. Acidolysis of **1b**

RP-HPLC linear gradients of H_2_O/MeCN (70:30) to (0:100) over 8 min.

#### 3.2.3. Acidolysis of **1c**

Compound **1c** (297.8 mg, 0.6 mmol) was treated with a solution of 1% TFA in MeCN (40 mL) during 28 h. After isolation, compound **2c** was obtained as a white solid (223.8 mg, 90% yield). RP-HPLC linear gradients of H_2_O/MeCN (70:30) to (0:100) over 8 min.

#### 3.2.4. Acidolysis of **1d**

RP-HPLC linear gradients of H_2_O/MeCN (70:30) to (0:100) over 8 min. Complete decomposition of **1d** was observed, compound **2d** was not detected.

#### 3.2.5. Acidolysis of **1e**

Compound **1e** (101.9 mg, 0.17 mmol) was treated with a solution of 1% TFA in MeCN (40 mL) during 15 h. After isolation, compound **2e** was obtained as a white solid (31.8 mg, 36% yield). RP-HPLC linear gradients of H_2_O/MeCN (70:30) to (0:100) over 8 min.

#### 3.2.6. Acidolysis of **1f**

RP-HPLC linear gradients of H_2_O/MeCN (70:30) to (0:100) over 8 min.

#### 3.2.7. Acidolysis of **1g**

Compound **1g** (400.7 mg, 0.75 mmol) was treated with a solution of 1% TFA in MeCN (40 mL) during 17 h. After isolation, compound **2g** was obtained as a white solid (287.7 mg, 85% yield). RP-HPLC linear gradients of H_2_O/MeCN (70:30) to (0:100) over 8 min.

#### 3.2.8. Acidolysis of **1h**

RP-HPLC linear gradients of H_2_O/MeCN (60:40) to (0:100) over 8 min. Complete decomposition of **1h** was observed (TFA 1% in MeCN), compound **2h** was detected by acidolysis treating **1h** with 1% TFA in MeCN/H_2_O (1:1), but suffer decomposition after 17 h of treatment.

#### 3.2.9. Acidolysis of **1i**

Compound **1i** (476.6 mg, 0.76 mmol) was treated with a solution of 1% TFA in MeCN (25 mL) during 48 h. After isolation, compound **2i** was obtained as a white solid (348.0 mg, 84% yield). RP-HPLC linear gradients of H_2_O/MeCN (50:50) to (0:100) over 8 min.

#### 3.2.10. Acidolysis of **1j**

Compound **1j** (100.0 mg, 0.11 mmol) was treated with a solution of 1% TFA in MeCN (25 mL) during 24 h. The reaction was quenched with Na_2_CO_3_ to pH = 7 and then the solvent was removed under reduced pressure. The obtained solid was automatically purified on a pre-packed Redisep Rf Gold C18 43 g column by using H_2_O/MeCN from 90:10 to 0:100 over 40 min. The fractions were collected and lyophilized obtaining compound **2j** as a white powder (44.9 mg, 70% yield). RP-HPLC linear gradients of H_2_O/MeCN (60:40) to (0:100) over 8 min.

#### 3.2.11. Acidolysis of **1k**

Compound **1k** (96.2 mg, 0.07 mmol) was treated with a solution of 1% TFA in MeCN (25 mL) during 24 h. The reaction was quenched with Na_2_CO_3_ to pH = 7 and the solvent was removed under reduced pressure. The obtained solid was automatically purified on a pre-packed Redisep Rf Gold C18 43 g column by using H_2_O/MeCN from 90:10 to 0:100 over 40 min. The fractions were collected and lyophilized obtaining compound **2k** as a white oily solid (74.5 mg, 86%). RP-HPLC linear gradients of H_2_O/MeCN (80:20) to (0:100) over 8 min.

#### 3.2.12. Acidolysis of **1l**

Compound **1l** (500.0 mg, 0.12 mmol) was treated with a solution of 1% TFA in H_2_O/MeCN (1:1) (50 mL) during 24 h. The reaction was quenched with NaHCO_3_ to pH = 7 and the MeCN was removed under reduced pressure. Extra water was added (40 mL) to the aqueous phase and then it was extracted with DCM (5 × 50 mL), the organic layer was dried over MgSO_4_ filtered and evaporated under reduced pressure. The obtained solid was automatically purified on a pre-packed Redisep Rf Gold C18 43 g column by using H_2_O/MeCN from 90:10 to 0:100 over 40 min. The fractions were collected and lyophilized obtaining compound **2l** as a white powder (316.3 mg, 70%). RP-HPLC linear gradients of H_2_O/MeCN (50:50) to (0:100) over 8 min.

#### 3.2.13. Acidolysis of **1m**

RP-HPLC linear gradients of H_2_O/MeCN (80:10) to (0:100) over 8 min. 

#### 3.2.14. Acidolysis of **1n**

RP-HPLC linear gradients of H_2_O/MeCN (60:40) to (0:100) over 8 min. Compound **1n** was stable into 1% TFA in MeCN.

## 4. Conclusions

The U-4CR has proved a powerful tool for the preparation of *N*-alkylated α,α-dialkylglycine amides, which show high steric tension due to the large number of bulky substituents present. This special scaffold facilitates C-terminal amide cleavage under acidic conditions. After preparing a short library subjected to different alkyl substitutions for the *N* and the α-carbon and also for the acyl, we performed a structure-acid lability relationship to identify those functional groups involved in amine release. Thus, it was demonstrated that both electron-donating effects in starting aromatic amines and flexible acyl chains (Gly) in the *N*-terminal favored the cis conformation. All these effects are related to the ease with which the oxazolonium ion intermediate forms and they promote the proximity of the central carbonyl group to the C-terminal amide, resulting in C-terminal amide cleavage. Furthermore, the introduction of cyclic compounds into dipeptide scaffolds also shown an increment in acidolysis rate and, consequently, amine release. Consistently, this structural acid lability relationship study should allow modulation of the conditions required for a given application. This *N*-alkylated α,α-dialkylglycine system should be of straightforward application in the design of new protecting groups and handles for solid-phase synthesis as is easily modulable and allow to fine tune it acid-lability.

## Figures and Tables

**Table 1:** Acidolysis results.

Acidolysis Study			
Entry	Starting Amide	t_1/2_ (Hours) ^a^	Yields (%) ^b^
**2a**	**1a**	32	36
**2b**	**1b**	5	90
**2c**	**1c**	3	100/90 ^c^
**2d**	**1d**	0 ^d^, 2 ^e^	decomp
**2e**	**1e**	4	95/36 ^c^
**2f**	**1f**	1.5	100
**2g**	**1g**	0.2	100/85 ^c^
**2h**	**1h**	0 ^d^, 3.4 ^e^	decomp.
**2i**	**1i**	0.1	100/84 ^c^
**2j**	**1j**	1.5	100/70 ^c^
**2k**	**1k**	0.4	100/86 ^c^
**2l**	**1l**	0 ^d^, 3.4 ^f^	100/70 ^c^
**2m**	**1m**	0.1	100
**2n**	**1n**	Stable	0

^a^ t_1/2_ was determined by the linearization of the acidolysis curve. ^b^ Corresponds to the chromatographic peak area (%) related to amide degradation at the indicated time. ^c^ Yield of isolated derivatives. ^d^ Complete product decomposition. Generally, acidolysis was performed using 1% TFA in MeCN, except for ^e^ acidolysis with 0.1% TFA in MeCN. ^f^ Acidolysis with 1% TFA in H_2_O/MeCN (1:1). Compound 1n was stable to 1% TFA in MeCN.

## Sample Availability

No new data were created or analyzed in this study. Data sharing is not applicable to this article.
